# ELISA-based detection of gentamicin and vancomycin in protein-containing samples

**DOI:** 10.1186/s40064-015-1411-y

**Published:** 2015-10-15

**Authors:** Jim C. E. Odekerken, Dorien M. W. Logister, Loubna Assabre, Jacobus J. C. Arts, Geert H. I. M. Walenkamp, Tim J. M. Welting

**Affiliations:** Laboratory for Experimental Orthopaedics, Department of Orthopaedic Surgery, Research School CAPHRI, Maastricht University Medical Centre, P.O. Box 5800, 6202 AZ Maastricht, The Netherlands

**Keywords:** ELISA, Gentamicin, Vancomycin, Drug release, Drug delivery

## Abstract

**Background:**

Orthopaedic implant infections are treated by surgical debridement, systematic antibiotic treatment or local antibiotic treatment with antibiotic-loaded beads. Currently antibiotic concentrations in wound exudate, serum, urine or tissue samples are determined with HPLC or fluorescent spectrometric assays. Both methods are heavily influenced due to proteins in the samples.

**Questions/purposes:**

Is ELISA capable to detect gentamicin and vancomycin in protein-containing samples like serum and wound exudate.

**Methods:**

Two specific competitive ELISA-assays were set-up to detect either gentamicin or vancomycin in protein-rich samples. An antibiotic-BSA hapten was generated as a coatable antigen and commercially available antibodies were applied for downstream immunodetection.

**Results:**

The developed ELISAs perform at a detection range of 2–500 ng/ml gentamycin and 20–5000 ng/ml vancomycin. Both ELISAs were capable of detecting these antibiotics in human serum and wound exudate without being compromised by the presence of proteins. We did not detect cross-reactivity for gentamicin in the vancomycin ELISA or vice versa.

**Conclusions:**

The antibiotic ELISAs detect gentamicin and vancomycin at low concentrations in protein-rich samples and they can be used as a high throughput and cost-effective alternative for chromatographic or fluorescent methods.

**Clinical relevance:**

These ELISAs can be used to detect very low gentamicin or vancomycin concentrations in clinical samples or assess novel orthopaedic antibiotic release systems in in vitro and in vivo studies.

**Electronic supplementary material:**

The online version of this article (doi:10.1186/s40064-015-1411-y) contains supplementary material, which is available to authorized users.

## Background

Orthopaedic infections are complex disorders, making prevention essential and treatment a challenging task (Calhoun et al. 2009; Miclau et al. 2010; Trampuz and Zimmerli 2006). Often the only possible treatment regimen to successfully treat a patient for orthopaedic infections is aggressive surgical debridement with intensive systemic antibiotic treatment, often supported by local antibiotic delivery (Calhoun et al. 2009; Zimmerli 2006; Zimmerli and Ochsner 2003). Gentamicin and vancomycin are two important antibiotics with a broad spectrum towards micro-organisms in severe orthopaedic infections (Fraimow 2009; Trampuz and Zimmerli 2006; Walenkamp et al. 1998). Gentamicin is an aminoglycoside-antibiotic and is mostly used to treat gram-negative species and *Staphylococci*, while vancomycin is a glycopeptide-antibiotic and is used against specific antibiotic-resistant strains of *Staphylococci* (Fraimow 2009; Trampuz and Zimmerli 2006; Walenkamp et al. 1998; Zimmerli 2006). These antibiotics are the most frequently used antibiotics admixed in polymethyl methacrylate (PMMA) bone cements for prosthesis fixation or as beads, both to serve as a local antibiotic delivery system in the prevention or treatment of orthopaedic infections (Wahlig et al. 1978; Walenkamp 2001; Walenkamp et al. 1998). Local antibiotic treatment is not only effective, but also avoids the toxicity of these antibiotics during systemic treatment such as ototoxicity and nephrotoxicity (Contreiras et al. 2014; Han et al. 2014; Nagai and Takano 2014; Ojano-Dirain et al. 2014; Walenkamp et al. 1986).

To follow the release of antibiotics from e.g. beads or spacers or determine systemic antibiotic concentrations in patient material (e.g. serum, urine, wound exudate and tissue samples), the quantification of the release of these antibiotics is essential (Wahlig et al. 1978). Initially these quantifications have been performed by fluorescent detection methods (Roche Diagnostics, approximate detection range 1–10 µg/ml) or chromatographic methods (e.g. high performance liquid chromatography (HPLC) with a minimal detection limit of 50 ng/ml) (Baietto et al. 2010; Manyanga et al. 2008; Wilson et al. 2003). In food and dairy applications gentamicin levels have been determined with enzyme-linked immunosorbent assays (ELISA) with more sensitive detection limits (as low as 1 ng/ml) (Haasnoot et al. 1999; Jin et al. 2005a, b) and only recently an ELISA-based method to measure vancomycin was published, (Chianella et al. 2013; Fujiwara et al. 2012). However, the use of such ELISA-based methods in human material has been minimally reported, possibly due to antibody restrictions and clinical diagnostic product regulations.

The goal of this study was to develop an indirect competitive ELISA-based detection method for gentamicin and vancomycin. In this setup a coated steady state antibiotic-hapten competes with the antibiotic in the sample for anti-antibiotic antibody binding. Due to this competition only antibodies bound to the steady state antibiotic-hapten will be detected by the conjugated secondary antibody, resulting in an HRP conjugate-dependent colorimetric signal which is inversely correlated to the antibiotic concentration in the sample. Therefore a low concentration of antibiotic in the sample will result in a high colorimetric absorbance value in the assay and vice versa.

See the Additional file 1: Figure S1 for a schematic representation of the ELISA setup.

To meet future requirements for (pre-) clinical use, our ELISA-based approach should be able to detect gentamicin and vancomycin in samples with a clinically relevant protein concentration and preferably in human serum and wound exudate as well.

## Methods

### Material collection, ethics and protein content

The used human serum originated from a single healthy volunteer. The collection of patient material (wound exudate) was approved by the Medical Ethics Committee of the Maastricht University Medical Centre (MEC approval number AZM/UM 11-4-023) and originated from a single patient.

Total protein concentration in human serum, human wound exudate (from hip revision surgery) and foetal calf serum (FCS, PAA Laboratories, Germany) was determined using the BCA method (Sigma-Aldrich, USA).

### Hapten preparation

Gentamicin sulphate (Sigma-Aldrich, USA) and vancomycin hydrochloride (Sigma-Aldrich, USA) were individually coupled to bovine serum albumin (BSA, PAA Laboratories, Germany) using N-(3-Dimethylaminopropyl)-N′-ethylcarbodiimide hydrochloride (EDC, Sigma-Aldrich, USA). The gentamicin-BSA hapten was prepared as follows: 50 mg BSA was dissolved in 1.5 ml phosphate buffered saline (PBS, pH 7.4) which was subsequently added drop-wise to 24.5 mg gentamicin sulphate. Three hundred milligram EDC was dissolved in 1 ml demineralized water and added drop-wise to the gentamicin-BSA mixture under continuous agitation. After 1 h incubation at room temperature the mixture was stored over night at 4 °C. The procedure for the vancomycin-BSA hapten preparation was comparable with the gentamicin-BSA hapten, only with 70 mg BSA in 18 ml PBS added to 77.8 mg vancomycin hydrochloride. The same amount of EDC was used only in 12 ml demineralized water. After 1 h incubation at room temperature the mixture was stored over night at 4 °C.

The combination hapten of both gentamicin and vancomycin with BSA was prepared using 33 mg BSA in 24 ml PBS added to 12 mg gentamicin sulphate and 36.7 mg vancomycin hydrochloride. Also 300 mg of EDC in 12 ml demineralized water was subsequently added drop-wise to the gentamicin-vancomycin-BSA mixture. After 1 h incubation at room temperature the mixture was stored over night at 4 °C.

After the 4 °C incubation step, uncoupled gentamicin and vancomycin as well as left-over EDC was removed from the preparation by dialysis (membrane cut-off value 12–14 kDa, Sigma-Aldrich, USA) at 4 °C over a period of 3 days with daily refreshment of the dialysis solution [phosphate buffered saline (PBS), pH 7.4]. After dialysis the haptens were stored in aliquots at −80 °C.

### Assessment of cross-linking of antibiotic compounds to BSA in hapten preparations

Polypeptides in hapten preparations were separated by sodium dodecyl sulphate polyacrylamide gel electrophoresis (SDS-PAGE) and transferred to polyvinylidene fluoride (PVDF, GE Healthcare Life Sciences, USA) membranes (for gentamicin-BSA, G-BSA) and nitrocellulose membranes (Protran BA 83, GE Healthcare Life Sciences, USA) (for vancomycin-BSA, V-BSA) by electroblotting. Immuno-blotting was performed with the same antibodies that are also used in the ELISA assay: mouse anti-gentamicin monoclonal antibody (clone 26.16, Abcam, USA) and rabbit anti-vancomycin polyclonal antibody (AbD Serotec, UK). Secondary antibodies conjugated with horseradishperoxidase (HRP) (Dako, Denmark) were used to assess the bound primary antibody fraction to the antibiotic-BSA haptens on the membranes which is subsequently visualized by enhanced chemiluminescence (ECL).

### Indirect competitive ELISA for gentamicin or vancomycin

The generated haptens were individually coated overnight at 4 °C to the surface of a 96-wells ELISA plate (10 ng coupled G-BSA, 1 µg V-BSA or 1.34 µg coupled G/V-BSA per well) in a 50 mM carbonate/bicarbonate buffer (pH 9.6). After incubation the plate was washed 3 times with wash-buffer [PBS/Tween-20 (0.05 % v/v)]. Subsequently wells were blocked [1 h incubation with wash-buffer/BSA (5 % wt./v)] Calibration curve samples were prepared in PBS/BSA (5 % wt./v) (range gentamicin: 0–1000 ng/ml and vancomycin: 0–5000 ng/ml) also the test samples (clinical isolates or serum spiked with a known concentration of antibiotics) were diluted 1000× in PBS/BSA before the initial measurement. Fifty microliter of the calibration curve sample or diluted test sample was pipetted in each well of the ELISA plate. Fifty microliter of diluted primary antibody (mouse anti-gentamicin monoclonal antibody (Abcam, USA), 7000× diluted in PBS/BSA; rabbit anti-vancomycin polyclonal antibody (Abd Serotec, UK), 5000× diluted in PBS/BSA) was added to the samples and incubated for 1 h at room temperature (conditions and antibody dilutions were based on experimental optimisation, data not shown). After incubation, the ELISA plate was washed 4 times with wash-buffer followed by an additional blocking step with wash-buffer/BSA (5 % wt./v) for 1 h. One-hundred microliter diluted secondary antibody (for gentamicin: rabbit anti mouse peroxidase (RAMPO, Dako, Denmark), 5000× diluted in PBS/BSA; for vancomycin: swine anti rabbit peroxidase (SWARPO, Dako, Denmark), 2000x diluted in PBS/BSA) was added to the wells and incubated for 1 h at room temperature after which the plate is washed 4 times with wash-buffer. After washing, 100 µl 3,3′,5,5′-tetramethylbenzidine (TMB, Sigma-Aldrich, USA) was added to each well to allow chromogenic detection of bound secondary antibodies. The reaction was stopped with 3 M sulphuric acid at the moment the absorbance of the negative control sample (0 ng/ml antibiotic) reached 0.5 at 650 nm. After stopping the reaction, the absorbance at 450 nm was measured using an ELISA reader (MultiSkan FC, Thermo Scientific). Due to the setup of the ELISA, the measured absorbance is inversely correlated with the antibiotic concentration present in the sample (for a detailed representation of the experimental procedure see Additional file 1: Figure S1). The antibiotic concentration present in the sample can be calculated based on the used calibration standards in a log–log scale.

### Regression analysis

Calibration curve fitment was calculated in Microsoft Excel 2010 by the use of the VBA Analysis ToolPak (Microsoft, USA). Graphical representation of the data was performed in GraphPad Prism 5 (GraphPad, USA).

## Results

### Protein concentration

Total protein concentration measurements of human serum, wound exudate or FCS showed that these contained 56, 46 and 37 mg total protein/ml, respectively. Based on these findings we decided to develop the ELISA assays in the presence of 50 mg/ml BSA to represent the protein concentration that generally occurs in the samples that we aim to measure.

### Hapten evaluation

**Fig. 1:**
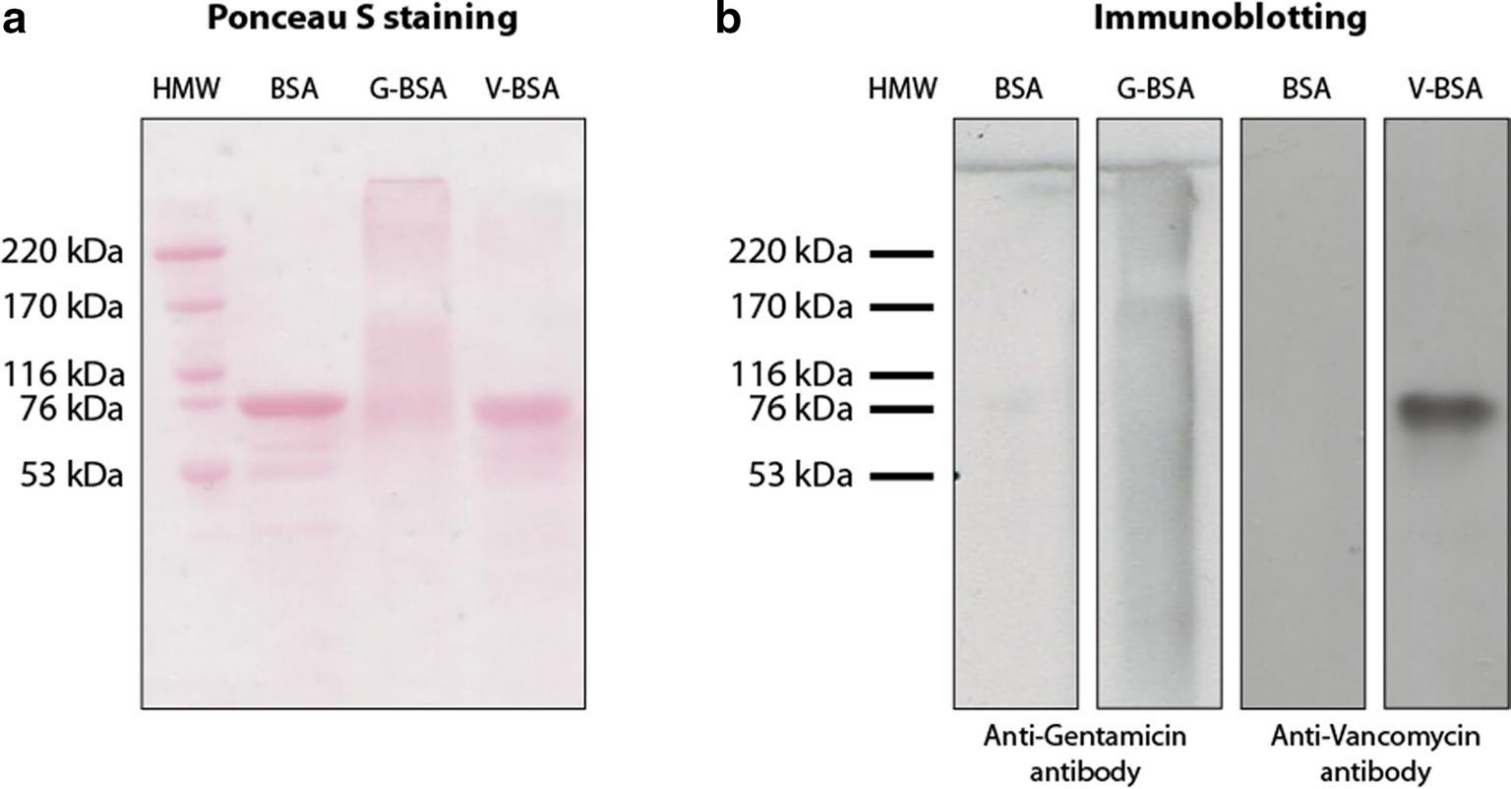
Hapten evaluation. **a** Ponceau S staining indicates protein presence in the coupled haptens. **b** Immunoblotting indicates corresponding antibiotic presence in both coupled haptens specifically

The development of these ELISAs required the coating of gentamicin or vancomycin to standard polystyrene ELISA wells. As the antibiotics themselves cannot be coated directly to well plates, we generated BSA haptens of each individual antibiotic by EDC-mediated coupling between a BSA carrier and the antibiotic. To acquire evidence of successful hapten generation we separated freshly prepared BSA-gentamicin and BSA-vancomycin haptens by SDS-PAGE, transferred the separated proteins to nitrocellulose membranes and performed anti-gentamicin or anti-vancomycin immunoblotting. To first visualize the total amount of SDS-PAGE-loaded hapten/protein, membranes were stained with Ponceau S after electro-blotting of SDS-PAGE gels. BSA runs at 66 kDa in SDS-PAGE and besides a major 66 kDa band, we were able to confirm the presence of ~140 kDa (BSA dimer) and ~210 kDa (BSA trimer) main bands, as well as a smear of higher molecular weight BSA species which were generated as a result of the EDC cross-linking (Fig. 1a). A band running at the size for BSA was found in the BSA-vancomycin haptene preparation, but no other higher molecular weight BSA species were detected by Ponceau S staining (Fig. 1a). To confirm the coupling of each individual antibiotic to BSA, antibiotic-specific immunodetection was performed. As presented in Fig. 1b, immuno-blotting against gentamicin specifically generated a signal in the lane loaded with the BSA-gentamicin hapten and not in the BSA-only lane. Similarly, a specific signal was detected in the BSA-vancomycin lane as compared to the BSA-only lane after anti-vancomycin immuno-blotting. These data show that we were successful in covalently coupling gentamicin or vancomycin to BSA for use as a hapten in the ELISA.

### Optimal hapten amount for coating of the microtiter plate

**Fig. 2:**
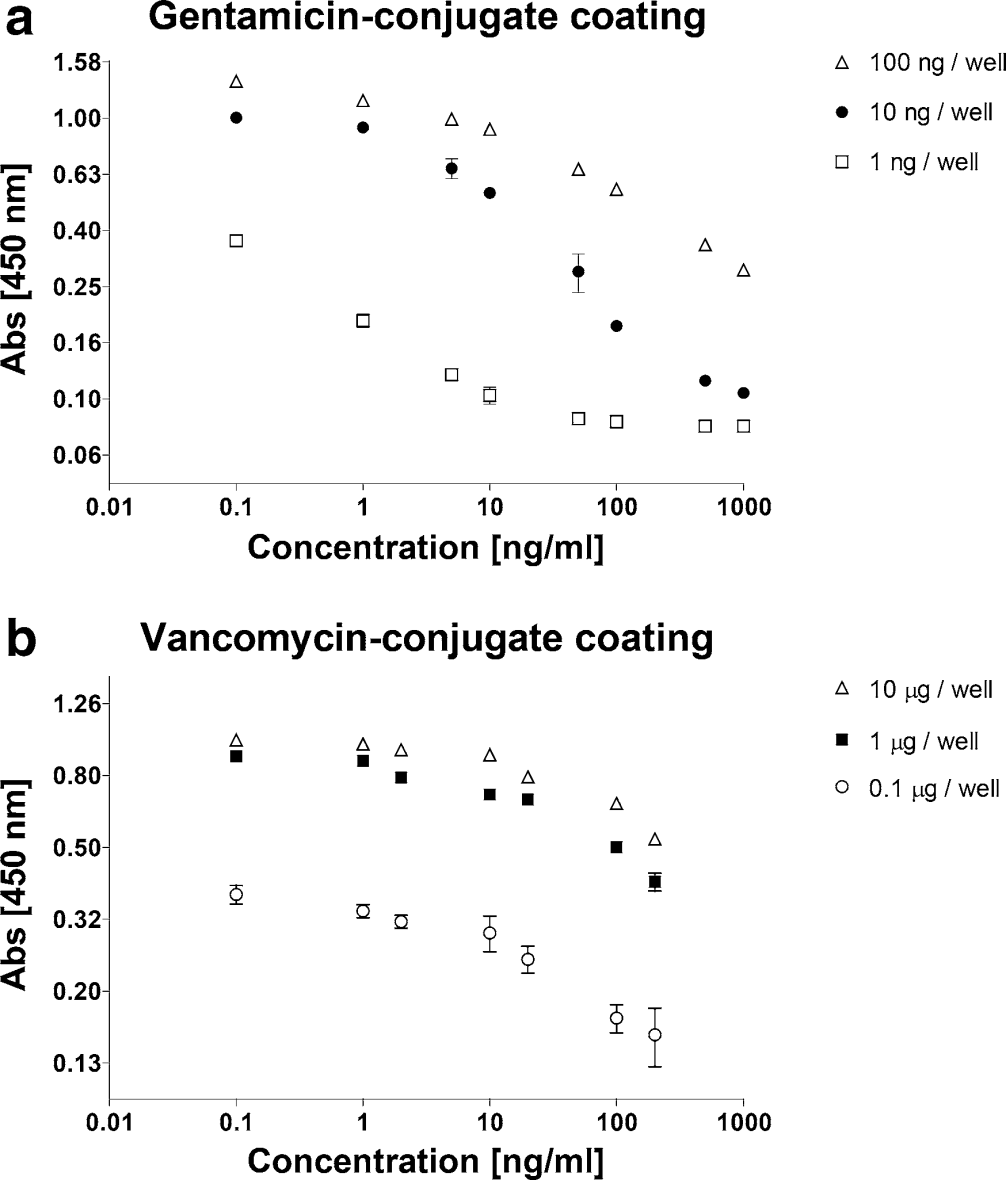
Coating optimization. **a** The optimal concentration of the gentamicin-BSA hapten was based on the pattern of the calibration curve, which indicated a concentration dependant absorbance. **b** The optimal concentration of the vancomycin-BSA hapten was determined on the relation between coating concentration and absorbance level

To determine the optimal coating amount for the hapten, three different hapten concentrations were coated on a microtiter plate. The gentamicin-BSA (G-BSA) hapten was coated at 1, 10 or 100 ng per well, while the vancomycin-BSA (V-BSA) hapten was coated at an amount of 0.1, 1 or 10 µg per well. Subsequently gentamicin or vancomycin detection properties of these amounts were evaluated. Performing the ELISA with a gentamicin concentration series indicated an inversely correlated gentamicin concentration-dependant signal in all G-BSA hapten coated wells. The 10 ng/well G-BSA hapten coating showed the broadest interpretable relation between gentamicin concentration and the A450 signal (Fig. 2a).

Performing the ELISA on the different hapten coating amounts with a vancomycin concentration series also indicated an inversely correlated vancomycin concentration-dependent signal in all V-BSA coated wells. Here, the 1 µg/well V-BSA hapten coating provided an optimal relation between the vancomycin concentration and the A450 signal. The 10 µg/well V-BSA haptene coating performed equally well and thus the 1 µg/well V-BSA became our condition of choice (Fig. 2b). From here on forward 10 ng/well G-BSA or 1 µg/well V-BSA were used to coat wells for any further experiments.

### Specificity and sensitivity of the gentamicin and vancomycin ELISAs

**Fig. 3:**
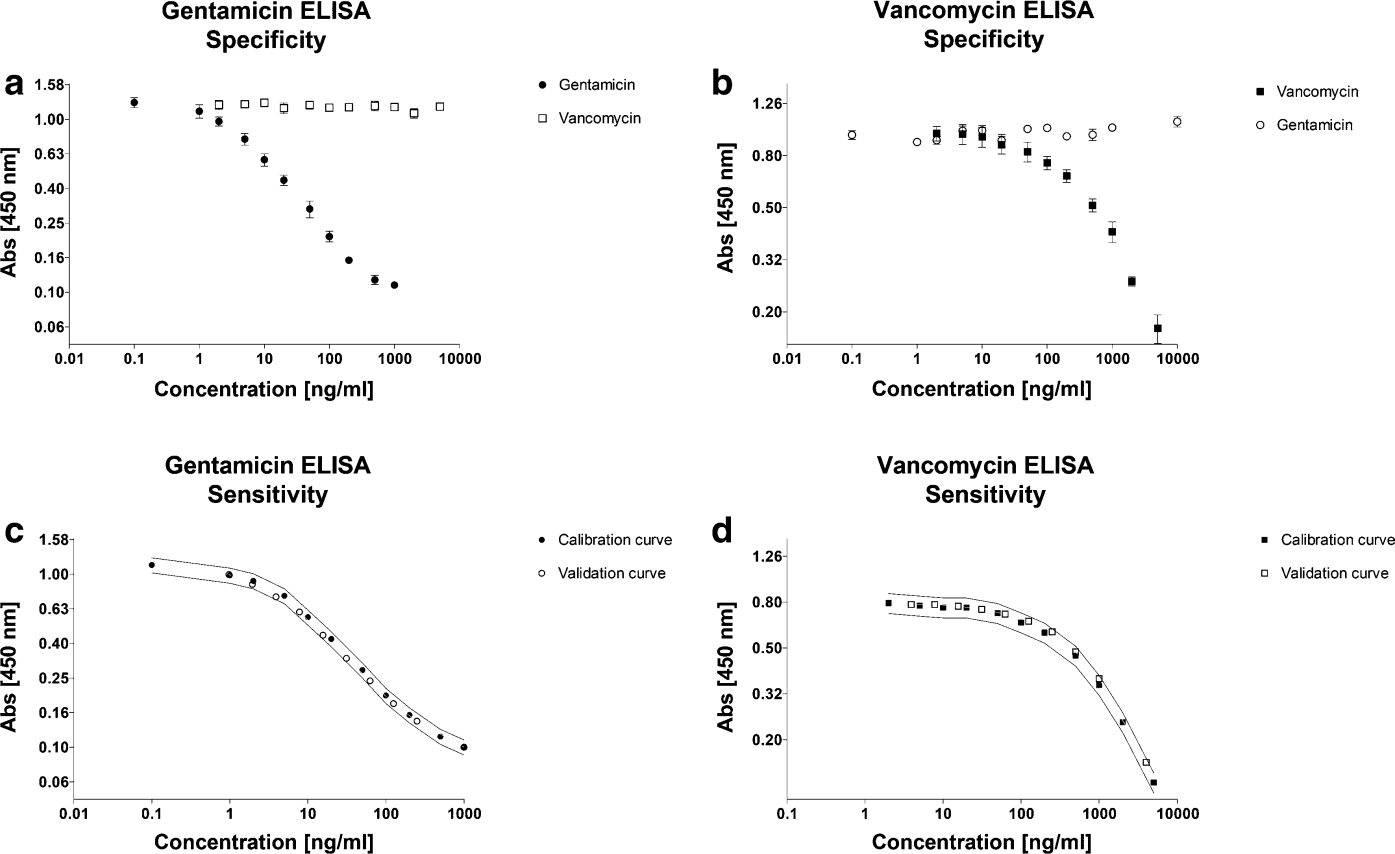
Specificity and sensitivity of the antibiotic ELISAs. **a** The influence of vancomycin on the gentamicin ELISA. **b** The influence of gentamicin on the vancomycin ELISA. **c** The validation of the calibration curve of the gentamicin ELISA. *Lines* indicate upper and lower 10 % range of the calibration curve. **d** The validation of the calibration curve of the vancomycin ELISA. *Lines* indicate upper and lower 10 % range of the calibration curve. *Error bars* indicate standard deviation

The specificity of the gentamicin ELISA was assessed by performing the gentamicin ELISA using a concentration series of either gentamicin or vancomycin. If the gentamicin ELISA would be aspecific for distinguishing gentamicin from vancomycin, it is expected that with increasing vancomycin concentrations a vancomycin-induced A450 shift would take place. The same principle was used for the vice versa situation where we determined the specificity of the vancomycin ELISA with a concentration series of gentamicin. The presence of vancomycin in the gentamicin ELISA did not result in a change of A450 absorbance in any of the tested concentrations, indicating that this ELISA setup is highly specific for gentamicin (Fig. 3a). These data also show that with this high specificity, the gentamicin ELISA is very sensitive and allows reliable detection of gentamicin in a range between 2 and 500 ng/ml. Gentamicin did not influence the vancomycin ELISA at any of the concentrations that were tested, indicating that also the vancomycin ELISA set-up is very specific for vancomycin (Fig. 3b). The detection range of the vancomycin ELISA was determined to be reliable between 20 and 5000 ng/ml vancomycin. To determine how the sensitivity of the ELISAs might depend on the antibiotic concentration in the sample, we used two separately prepared antibiotic concentration series. One series was used to generate a calibration curve and the other concentration series (“validation series” in the Figure) was subsequently measured and the antibiotic concentration in the series was estimated by using the calibration curve. This was done for both ELISAs separately. As shown in Fig. 3c, d we found that for both ELISAs the samples in the validation series generated absorbances that were within the 10 % deviation range of the measured absorbance of the calibration curve (often used in commercial kits, to compensate for potential pipetting errors, differences between wells and the standard deviation of the measurements) (Fig. 3c, d).

### Antibiotic concentration in high-protein samples

In an orthopaedic context, the gentamicin and vancomycin ELISAs are expected to be used, amongst others, for determining antibiotic concentrations in clinical samples such as wound exudate from post-surgical drainage. To determine the performance of our ELISAs in this context, human wound exudate (total protein concentration was 56.8 mg/ml, see above) was spiked with vancomycin to a final concentration of 50 µg/ml, to simulate a sample acquired from a patient undergoing antibiotic treatment. Before performing the ELISA, the sample was pre-diluted (1000×) in PBS/BSA. The vancomycin ELISA showed that the sample contained 53.5 µg/ml vancomycin, indicating a recovery of 107 %. The wound exudate was not spiked with gentamicin since the patient from whom the exudate was acquired was under gentamicin treatment. However this offered us the opportunity to determine whether we could detect the presence of gentamicin in the patient’s wound exudate. Using our gentamicin ELISA we found that the wound exudate contained 5.0 µg gentamicin/ml. In addition we spiked human serum with gentamicin to a final concentration of 5 µg/ml to simulate a sample acquired from a patient undergoing antibiotic treatment. After initial sample dilution (1000×) the gentamicin ELISA determined that the serum sample contained 4.5 µg gentamicin/ml, indicating a recovery of 90 %. Also, concentration series of gentamicin (Table 1) and vancomycin (Table 2) in PBS/BSA indicated recovery ranges between 91.6 and 108.8 %.

**Table 1:** Accuracy gentamicin ELISA

Gentamicin concentration in validation series (ng/ml)	Concentration determined by linear regression (ng/ml)	Recovery (%)
7.8	8.3	105.8
15.6	16.8	107.5
31.3	33.9	108.4
62.5	67.3	107.7
125.0	134.9	107.9
250.0	230.2	92.1
Human serum (5 ng/ml)	4.5	90.0

**Table 2:** Accuracy vancomycin ELISA

Vancomycin concentration in validation series (ng/ml)	Concentration determined by polynomial regression (ng/ml)	Recovery (%)
31.3	33.0	105.6
62.5	68.0	108.8
125.0	114.5	91.6
250.0	258.6	103.4
750.0	766.3	102.2
4000.0	4282.1	107.1
Hum. wound exudate (50 ng/ml)	53.5	107.0

### Multiple conjugates in one hapten

**Fig. 4:**
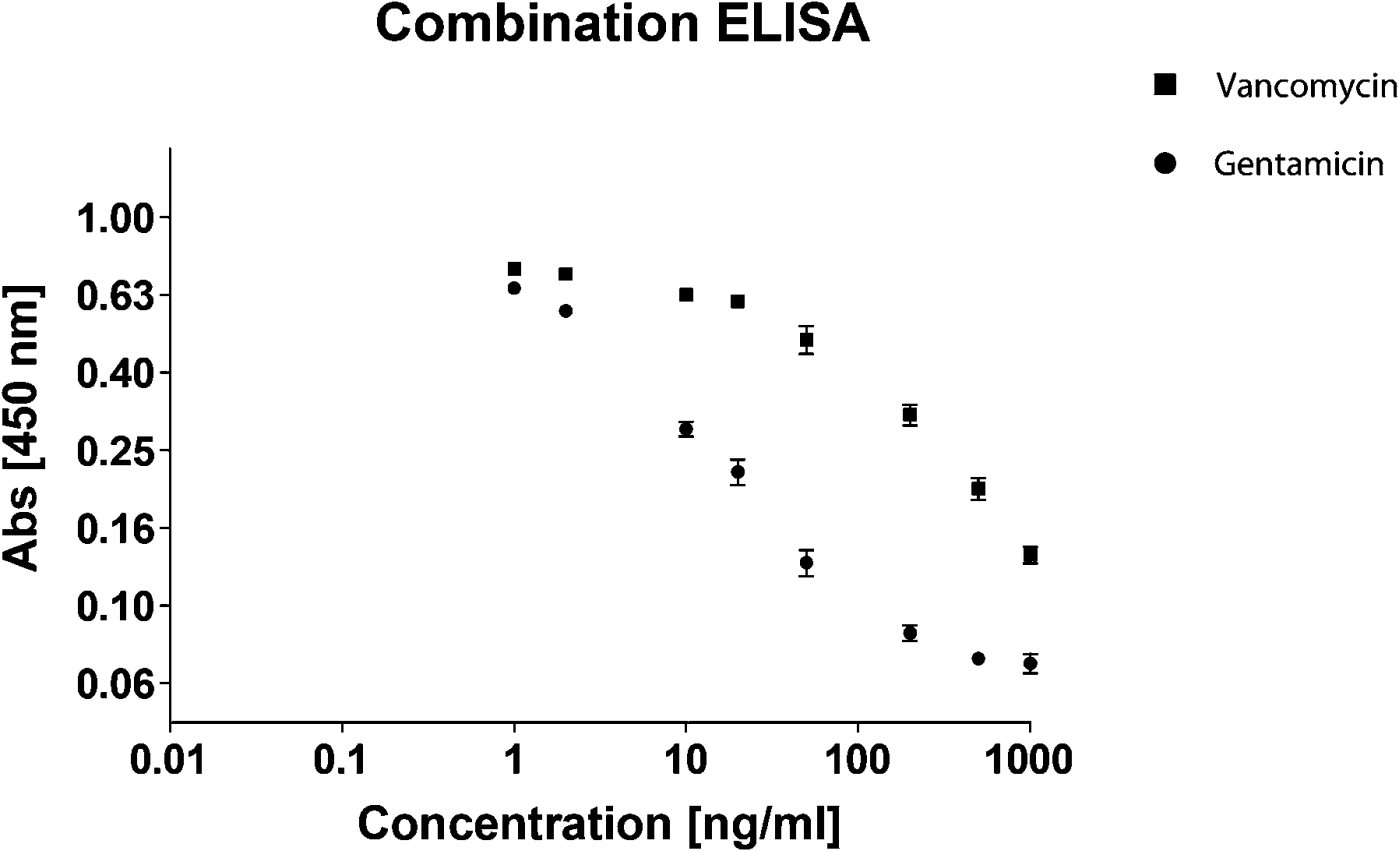
ELISA for gentamicin and vancomycin. A combined hapten of both gentamicin and vancomycin was used to coat wells for the detection of either gentamicin (*black circles*) or vancomycin (*black squares*) in a calibrations series of the respective antibiotics. *Error bars* indicate standard deviation

By using BSA as a coatable protein for generating the gentamicin and vancomycin haptens, we explored the possibility of combining different antibiotics in one hapten coupling reaction. This potentially offers the opportunity to generate a combined assay for the detection of both antibiotics by using the same hapten in separate dedicated ELISAs. A combined hapten of both gentamicin and vancomycin cross-linked to BSA indeed allowed the detection of either gentamicin or vancomycin by their respective antibodies, in a comparable range as the individual ELISA protocols (Fig. 4). Furthermore, when this hapten was used for the ELISA detection of gentamicin, it was not influenced by vancomycin presence in the sample and vice versa.

## Discussion

Orthopaedic infections are complex disorders often requiring surgical treatment and implantation of a local antibiotic delivery system. The use of antibiotic containing PMMA bone cement, beads or spacers is considered effective to prevent and treat such infections in combination with systemic antibiotics (Geurts 2011; Klemm 1979; Wahlig 1972; Wahlig et al. 1978; Walenkamp et al. 1998). Gentamicin beads and spacers generally remain implanted in the patient for a couple of weeks, before being surgically removed and substituted by a prosthesis or osteosynthesis materials (Geurts 2011; Walenkamp et al. 1998).

Currently HPLC and fluorescence-based methods are used to detect antibiotics in patient material (Baietto et al. 2010; Manyanga et al. 2008; Wilson et al. 2003). These methods often require sample pre-treatments, are expensive, often insensitive or prone to be influenced by the high protein content in (patient) sample material (Baietto et al. 2010; Manyanga et al. 2008; Wilson et al. 2003). Currently, these methods are the method of choice to assess novel antibiotic release systems and coatings for clinical use. ELISA-based methods have been sparsely used for this purpose, possibly due to the lack of ready-to-use protocols for ELISA-based antibiotic detection methods. ELISA-based methods are generally cheaper per sample, and better suited for high-throughput applications, compared to HPLC.

Based on protocols derived from dairy and food-industry applications we established ELISA-based assays to determine the concentration of gentamicin and vancomycin in patient material (serum and wound exudate). The gentamicin ELISA has a reliable detection range of 2-500 ng/ml with no detectable cross-reactivity with vancomycin. The vancomycin ELISA is reliable between 20 and 5000 ng/ml without cross-reactivity with gentamicin. Due to the graph characteristics of the concentration-dependent calibration curve of gentamicin a linear regression can be used to estimate the concentration in unknown samples. For vancomycin a linear regression is not preferable due to the bending of the curve. Therefor a polynomial regression was used to estimate the vancomycin concentrations of unknown samples. The sensitivity of both ELISA’s is high, especially when compared to the detection limits of the frequently used fluorescence-based detection systems and when compared to HPLC, where high-protein content is a notoriously hampering factor (Haasnoot et al. 1999; Jin et al. 2005a, b; Wilson et al. 2003). The accuracy of the ELISA’s however will depend on the composition and complexity of the samples, therefore a possible optimization step may be required before specific application of the assay for quantitative measurements. Still in this proof of concept we have shown that the ELISA’s are sensitive enough to allow accurate estimation of the antibiotic concentrations in “unknown”/spiked samples containing biologically common protein concentrations.

Recently novel experimental approaches to detect gentamicin and vancomycin have been reported in literature (Chianella et al. 2013; Fujiwara et al. 2012). The group of Chianella recently described the use of a specific synthetic coatable molecularly imprinted polymer nanoparticle (nanoMIP) for the detection of vancomycin in an ELISA-like way, instead of a conventional antibiotic-protein hapten (Chianella et al. 2013). Very low concentration ranges (pM) are achieved by using the high-tech nanoMIP method. However, depending on the relevant concentrations to be measured, our low-tech ELISA setup provides an easy accessible and low-cost method for an in-house generated ELISA assay in the nM range. The group of Fujiwara described the use of a hapten consisting of an antibiotic cross-linked to a protein like BSA. Only they choose a different cross-linking agent resulting in an antibiotic-cross-linker-BSA hapten (Fujiwara et al. 2012). In our approach we use a zero-length cross-linker (EDC) which results in an antibiotic-BSA hapten without the cross-linker in the hapten. A possible interaction of a cross-linker in the assay is thereby avoided.

Our data shows that the gentamicin ELISA requires a lower amount of hapten to be coated to the surface of the microtiter plate well as compared to the vancomycin ELISA. This difference might be related to the size of the individual antibiotic compounds. Gentamicin is a relatively small molecule in comparison to vancomycin (about 1/3 in molecular weight). To achieve the coating of an equimolar amount of vancomycin as compared to gentamicin, it is imperative that more V-BSA hapten is coated to the well. However this does not fully explain the 100-fold larger coating dose required for the vancomycin ELISA. In addition this could also be attributed by a difference in cross-linking efficiency during hapten formation, as seen in the immunoblotting results where the cross-linking of gentamicin resulted in BSA di-mers and tri-mers. Furthermore, the applied individual antibodies for the detection of gentamicin and vancomycin are different and will differ in their antibody-antigen binding affinity. This difference in antibodies might thus influence the detection ranges in the assays and provide an explanation why we need to coat more V-BSA hapten to the well than G-BSA hapten. Although the gentamicin antibody does not detectably interact with vancomycin and the vancomycin antibody does not interact with gentamicin, we cannot exclude that other antibiotics might cause aspecific interactions with the herein described antibodies, potentially resulting in incorrect calculations of the antibiotic concentration in the measured sample. Future application-specific evaluation of this potential bias should be performed, depending on the antibiotic.

Due to the nature of our ELISA setup, the protocol can be easily converted to establish ELISA tests for other antibiotics, provided the availability of antibodies and that such antibiotics possess carboxyl-groups or primary amines (like tobramycin, kanamycin, sisomicin and cefuroxime). This is due to the use of EDC as a cross-linking agent to establish the antibiotic-BSA hapten. The versatility of this ELISA setup also allowed the detection of both vancomycin and gentamicin by one hapten source, in which the two different antibiotics were combined. From an efficiency point-of-view this is particularly interesting, since only one hapten has to be produced to allow detection of a variable set of antibiotics.

## Conclusion

We here describe an easy-to-use protocol for the detection of gentamicin and vancomycin by ELISA. The protocol can be adapted relatively easy to other antibiotics, enabling the detection of antibiotics from experimental and clinical antibiotic release studies in several types of liquids, including high-protein samples.

## Additional file

10.1186/s40064-015-1411-y Schematic representation of the antibiotic ELISA procedure and detection. The antibiotic-BSA hapten is coated to a microtiter plate. An antibody directed against the antibiotic compound can interact with either the antibiotic in the sample or the antibiotic-hapten coated to the microtiter plate. After washing only the microtiter plate-bound antibodies remain present and can be detected by HRP-conjugated secondary antibodies in combination with TMB (colorimetric detection at 450 nm). A high concentration of antibiotics in a sample will lead to more interaction with an antibiotic-specific antibody in solution and thus result in less bound antibodies to the microtiter plate leading to a low signal when detected at 450 nm. This indicates that the measured signal is inversely correlated with the concentration of antibiotic in the measured sample. Samples with a known concentration of antibiotics can be used for a calibration curve; regression from this curve will allow calculation of the antibiotic concentration in unknown samples.
