# Supplementary figures and images for: ELISA-based detection of gentamicin and vancomycin in protein-containing samples
Source: Springerplus. 2015 Oct 15;4:614. doi: 10.1186/s40064-015-1411-y (PMC4628023; doi:10.1186/s40064-015-1411-y)

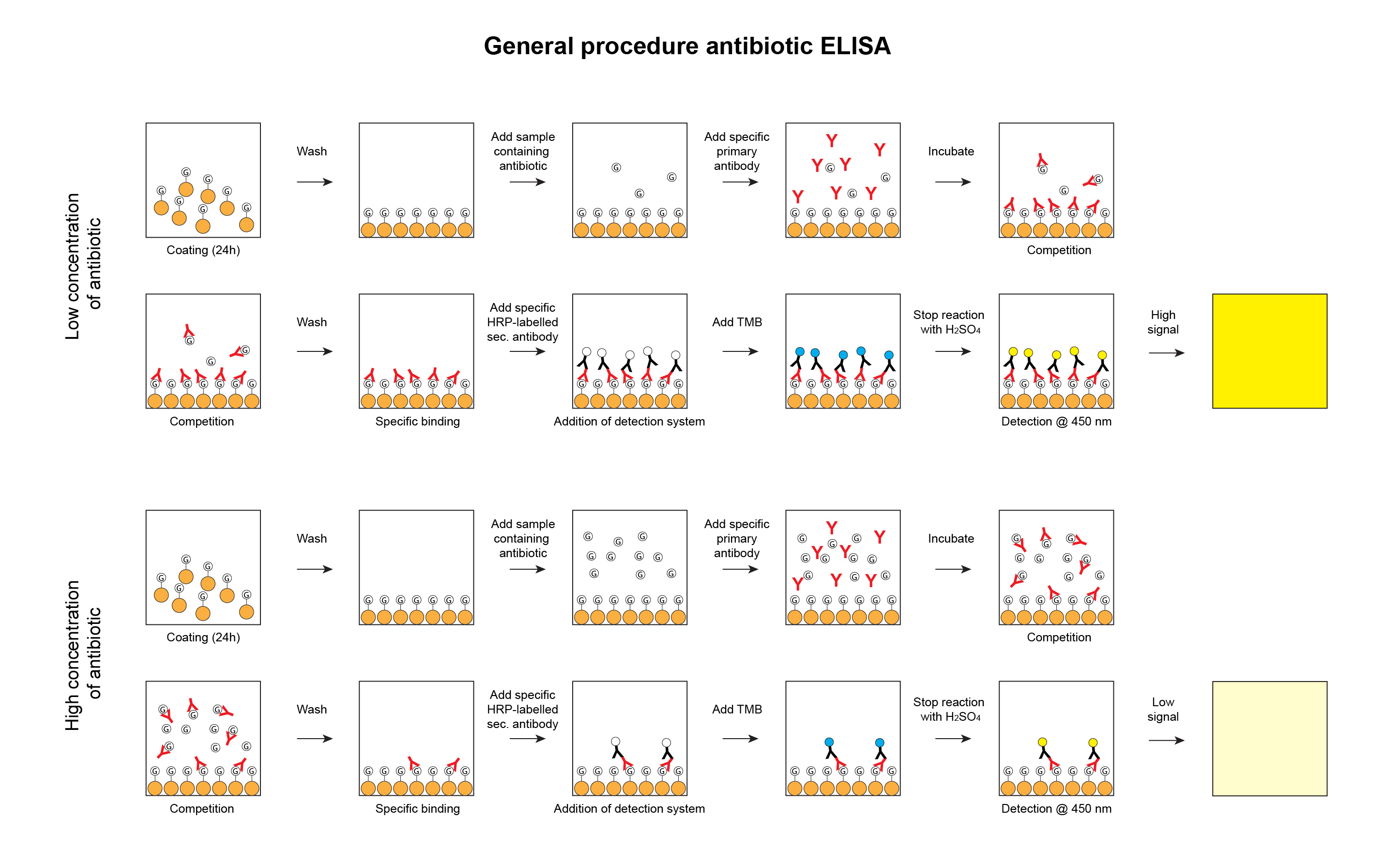

Supplement: Supplementary file 1 — 10.1186/s40064-015-1411-y Schematic representation of the antibiotic ELISA procedure and detection. The antibiotic-BSA hapten is coated to a microtiter plate. An antibody directed against the antibiotic compound can interact with either the antibiotic in the sample or the antibiotic-hapten coated to the microtiter plate. After washing only the microtiter plate-bound antibodies remain present and can be detected by HRP-conjugated secondary antibodies in combination with TMB (colorimetric detection at 450 nm). A high concentration of antibiotics in a sample will lead to more interaction with an antibiotic-specific antibody in solution and thus result in less bound antibodies to the microtiter plate leading to a low signal when detected at 450 nm. This indicates that the measured signal is inversely correlated with the concentration of antibiotic in the measured sample. Samples with a known concentration of antibiotics can be used for a calibration curve; regression from this curve will allow calculation of the antibiotic concentration in unknown samples. [file 40064_2015_1411_MOESM1_ESM.jpg]
